# Editorial: Stress tolerance in sorghum: molecular mechanisms, gene discovery, and quality dynamics

**DOI:** 10.3389/fpls.2026.1938261

**Published:** 2026-09-22

**Authors:** Peng Lv, Guoquan Liu, Jieqin Li

**Affiliations:** 1 Institute of Millet Crops, Hebei Academy of Agricultural and Forestry Sciences, Shijizhuang, China; 2 The University of Queensland, Brisbane, QLD, Australia; 3 Anhui University of Technology, Maanshan, China

**Keywords:** abiotic, advances, biotic, sorghum, stress

Global agriculture is confronted with compounded climate crises: frequent droughts, soil salinization, waterlogging, rising pathogen and insect pressure, and a growing demand for eco-friendly grain production systems. As the world’s fifth most vital cereal crop, Sorghum bicolor L. Moench stands out as a climate-smart crop uniquely equipped to sustain food security in arid, semi-arid, and marginal agroecosystems. This special thematic collection assembles ten original research and review articles that systematically advance sorghum stress biology, green disease management, translational genomics, and modern biotechnological breeding. As the three chief editors of this journal, we hereby contextualize the collective value of these contributions, highlight cross-cutting research breakthroughs, and outline critical future directions for sorghum sustainable agriculture.

Climate change-driven abiotic stresses—drought, salinity, and waterlogging—are the primary yield-limiting barriers for sorghum cultivation worldwide, and six papers in this Research Topic dissect the morphological, physiological, and genetic regulatory networks governing sorghum stress tolerance. Drought is the most pervasive water limitation for cereal production, and sorghum’s innate drought hardiness makes it an ideal model for monocot stress research. One Article comprehensively reviews omics tools, drought-resistance gene regulatory pathways, and the advantages of sorghum over model dicots for climate-resilient crop research, while acknowledging persistent bottlenecks in genetic transformation efficiency. Complementarily, Another article performs a genome-wide association study (GWAS) on a global mini-core sorghum landrace panel under polyethylene glycol-simulated seedling drought, identifying 22 tolerance loci and 19 candidate genes, and pinpointing two extreme drought-tolerant and sensitive germplasm accessions for mechanistic validation.

Soil salinization devastates sorghum seedling establishment across coastal and inland saline farmlands, addressed by two complementary GWAS and germplasm evaluation studies. One study uses 245 mini-core accessions and over six million high-quality SNPs to map 35 salt-tolerance genomic loci and 39 candidate genes associated with ion transport and stress signaling, laying a foundation for marker-assisted breeding. Another paper expands germplasm resources by phenotyping 188 grain sorghum lines under 150 mM NaCl, establishing a multi-index comprehensive salt-tolerance evaluation system based on shoot and root biomass traits. The work confirms that tolerant sorghum accumulates higher proline, soluble sugars, and antioxidant enzyme activity (SOD, POD, CAT), with lower malondialdehyde oxidative damage, providing elite salt-tolerant germplasm LCS177 and LCS234 for breeding pipelines. Beyond genetic dissection, one paper delivers a practical exogenous regulation strategy: hydrogen sulfide (H_2_S) application activates the chloroplast AsA-GSH antioxidant cycle, stabilizes photosystem II integrity, and alleviates salt-induced oxidative injury in sorghum seedlings, offering a chemical priming approach for saline field production.

Waterlogging represents another understudied critical abiotic constraint for sorghum planted in rainy lowland regions. One study contrasts a waterlogging-tolerant genotype S208 and sensitive line S015, revealing that superior tolerance stems from coordinated morphological adaptations (adventitious root proliferation, aerenchyma formation), elevated anaerobic respiratory enzyme activity (PDC, LDH, ADH), and transcriptional reprogramming mediated by AP2/ERF, BHLH, and WRKY transcription factors. This study clarifies the multi-layered adaptive machinery of sorghum under hypoxic conditions, filling gaps in sorghum waterlogging molecular physiology. To deepen transcription factor-mediated stress regulatory research, a article conducts a genome-wide identification of the conserved NF-YA transcription factor family in sorghum, characterizing nine SbNF-YA genes and verifying their drought-responsive expression patterns via qRT-PCR, expanding the repertoire of core regulatory genes for sorghum abiotic stress improvement.

Beyond abiotic stress resilience, two articles tackle biotic threats to sorghum production, covering eco-friendly disease biocontrol and aphid pest resistance mechanisms—two urgent priorities for organic sorghum supply chains, especially sorghum cultivated for Chinese Baijiu brewing. One article evaluates six biofungicides and two microbial agents against Colletotrichum sublineola, the causal agent of sorghum anthracnose. *In vitro*, greenhouse, and multi-site field trials consistently validate pterostilbene and Bacillus subtilis as high-efficiency biocontrol agents, with field efficacy exceeding single microbial fungi (Trichoderma harzianum). This work delivers low-environmental-risk microbial formulations to reduce chemical fungicide reliance, addressing the acute shortage of commercial biocontrol products for organic sorghum. For insect stress, Another article reviews sorghum interactions with two destructive aphid pests (Melanaphis sacchari, Schizaphis graminum), synthesizing resistance QTL mapping, molecular defense mechanisms, and integrated pest management frameworks. The authors emphasize that genome editing and transgenic molecular breeding will accelerate the development of aphid-resistant sorghum varieties to counter evolving pest virulence under climate warming.

One article serves as a capstone review integrating all stress research themes, systematically summarizing transformative biotechnological innovations for sorghum improvement: genotype-independent transformation using WUS2/BBM, RNAi grain quality modification, transgene-free CRISPR genome editing, nanobiotechnology, and synthetic apomixis. It underscores that stacking multi-stress resistance traits via precision genome manipulation is the definitive path to develop sorghum varieties adapted to compound climate stresses, bridging basic stress genetics and applied breeding.

Collectively, this Research Topic forms a complete research chain spanning germplasm screening, physiological phenotyping, multi-omics gene discovery, transcription factor functional verification, exogenous chemical regulation, microbial biocontrol, and cutting-edge genetic engineering. The ten papers deliver dual values: fundamental mechanistic insights into sorghum stress response, and actionable field technologies for organic and climate-smart sorghum cultivation. For industrial relevance, the biocontrol research targeting anthracnose directly supports the booming organic sorghum raw material industry for Baijiu, creating circular agricultural solutions that balance economic output and ecological protection.

Nevertheless, as editors, we identify three key gaps requiring concerted research effort moving forward. First, most current experiments focus on seedling-stage stress responses; field-based whole-life-cycle stress trials under combined drought-salt-waterlogging co-stress remain scarce, limiting translation of lab findings to commercial production. Second, functional validation of the hundreds of candidate stress genes identified via GWAS and transcriptomics lags far behind mapping work, and few genes have been deployed in commercial sorghum breeding programs. Third, integrated management strategies combining microbial biocontrol, exogenous stress priming, and genetic resistance need field-scale optimization to build full-spectrum stress-resilient sorghum production systems.

We anticipate that the findings published in this thematic issue will stimulate cross-disciplinary collaboration between plant physiologists, geneticists, microbial biologists, and crop breeders. By harnessing sorghum’s innate stress tolerance and advancing biotechnological toolkits, researchers can accelerate the delivery of multi-stress-resistant, high-yield, high-quality sorghum varieties to smallholder and large-scale producers across marginal agroecosystems. In an era of mounting global food insecurity, sorghum will continue to rise as an irreplaceable strategic cereal, and we are proud to present this body of rigorous, solution-oriented research to advance its sustainable development.

